# Organic-Inorganic Hybrid Planarization and Water Vapor Barrier Coatings on Cellulose Nanofibrils Substrates

**DOI:** 10.3389/fchem.2018.00571

**Published:** 2018-11-21

**Authors:** Feyza Karasu, Luca Müller, Hassan Ridaoui, Mohammed Ibn ElHaj, Göran Flodberg, Christian Aulin, Lars Axrup, Yves Leterrier

**Affiliations:** ^1^Laboratory for Processing of Advanced Composites (LPAC), École Polytechnique Fédérale de Lausanne (EPFL), Lausanne, Switzerland; ^2^Rolic Technologies Ltd., Allschwil, Switzerland; ^3^RISE Bioeconomy, Stockholm, Sweden; ^4^Stora Enso Karlstad Research Centre, Karlstad, Sweden

**Keywords:** cellulose nanofibrils, diffusion barrier, hybrid materials, multilayers, packaging

## Abstract

Cellulose nanofibrils (CNF) can be produced in the form of thin, transparent and flexible films. However, the permeability of such materials to oxygen and water vapor is very sensitive to moisture, which limits their potential for a variety of packaging and encapsulation applications. Diffusion barrier coatings were thus developed to reduce the access of water molecules to enzymatically pre-treated and carboxymethylated CNF substrates. The coatings were based on UV curable organic-inorganic hybrids with epoxy, tetraethylorthosilicate (TEOS) and 3-glycidoxypropyltrimethylenesilane (GPTS) precursors and additional vapor formed SiN_x_ layers. A total of 14 monolayer and multilayer coatings with various thickness and hybrid composition were produced and analyzed. The water vapor transmission rate (WVTR) of the bilayer epoxy/CNF film was two times lower compared to that of uncoated CNF film. This was partly due to the water vapor permeability of the epoxy, a factor of two times lower than CNF. The epoxy coating improved the transparency of CNF, however it did not properly wet to the CNF surfaces and the interfacial adhesion was low. In contrast hybrid epoxy-silica coatings led to high adhesion levels owing to the formation of covalent interactions through condensation reactions with the OH-terminated CNF surface. The barrier and optical performance of hybrid coated CNF substrates was similar to that of CNF coated with pure epoxy. In addition, the hybrid coatings provided an excellent planarization effect, with roughness close to 1 nm, one to two orders of magnitude lower than that of the CNF substrates. The WVTR and oxygen transmission rate values of the hybrid coated CNF laminates were in the range 5–10 g/m^2^/day (at 38°C and 50% RH) and 3–6 cm^3^/m^2^/day/bar (at 23°C and 70% RH), respectively, which matches food and pharmaceutical packaging requirements. The permeability to water vapor of the hybrid coatings was moreover found to decrease with increasing the TEOS/GPTS ratio up to 30 wt% and then increase at higher ratio, and to be much lower for thinner coatings due to further UV-induced silanol condensation and faster evaporation of byproducts. The addition of a single 150 nm thick SiN_x_ layer on the hybrid coated CNF improved its water vapor barrier performance by more than 680 times, with WVTR below the 0.02 g/m^2^/day detection limit.

## Introduction

The transport of gases through food and pharmaceutical packaging is often not desirable as it may lead to damaged and spoiled goods. Glass and aluminum provide perfect barriers, but are either brittle, or opaque and associated with a large carbon footprint compared with polymers such as PET (Simon et al., 2016). These concerns led to the development of a broad diversity of gas-barrier polymer based materials, mostly in the form of multilayer films to fulfill the many requirements for food and pharma products. Target permeance values (or gas transmission rates) for these applications are of the order of few cm^3^/m^2^/day/bar for oxygen, and few g/m^2^/day for water vapor (Lange and Wyser, 2003). Such permeation levels were obtained using intrinsically high barrier polymers and nanocomposites (Wagner, 2016). Alternative vapor formed dielectric films deposited onto semi-crystalline polymers were developed in the 1990s, leading to a 100-fold reduction of the permeance of the bare polymer substrate (Chatham, 1996), the residual permeation being controlled by defects in the film (Leterrier, 2003). Similarly, the ingress of water through the encapsulation layers of flexible electronic and displays devices such as organic light emitting diodes (OLED) severely impact the lifetime of the devices (Nisato et al., 2001). In these applications target permeance values are far below those for food packaging. In the case of OLED, these are typically 10^5^-10^6^ times lower, for both oxygen and water, to guarantee expected lifetimes of few years. This highly demanding performance stimulated considerable research efforts in the last two decades to develop flexible ultrahigh barrier films (Kim et al., 2004; Priolo et al., 2010; Yang et al., 2011; Fahlteich et al., 2013; Gokhale and Lee, 2014; Guin et al., 2014). The most successful are presently in the form of multilayer organic-inorganic stacks deposited onto optically transparent thermoplastic polyester substrates, that are moreover compatible with roll-to-roll processes (Vaško et al., 2009; Fahlteich et al., 2014; Yu et al., 2016). The organic interlayers enable to decouple the influence of defects in adjacent inorganic layers and considerably increase the diffusion path. Typically, up to 2–3 inorganic layers, that is, a total of 5 layers or more are required to achieve the above-mentioned low permeation levels. Notice that an adequate surface planarization step of the polymer substrate, such as the application of an hybrid organic-inorganic ‘hardcoat’ layer is usually required to improve the quality of the first inorganic layer, leading to barrier improvement factors of 100 or more with respect to the plain, uncoated polymer substrate (Affinito et al., 1996; Coclite and Gleason, 2012; Fahlteich et al., 2013). A key challenge for ultrahigh barriers is to reduce the number of layers hence the production cost of the barrier coating without losing performance. Attempts to this end include atomic layer deposition (Gokhale and Lee, 2014) and the creation of films with a graded composition (Choi et al., 2010). Additional outstanding limitations of these technologies remain the brittleness of the inorganic layers, with a strain at failure around 1% (Leterrier, 2015), their energy and cost intensive production and the use of synthetic polymer substrates. There is, therefore, and in addition to cost reduction and permeability reduction, a growing demand for more environmentally friendly barrier materials (Vartiainen et al., 2016).

A promising alternative to conventional oil-based polymers for packaging and encapsulation materials is cellulose nanofibrils (CNF) (Zhang et al., 2013; Nair et al., 2014; Vaha-Nissi et al., 2017). The environmental impact of CNF production, especially via the enzymatic route, is indeed lower than that of synthetic polymer materials (Amienyo et al., 2013; Li et al., 2013; Arvidsson et al., 2015). CNF is a hydrogen-bonded network of stiff and straight fibrils with a high crystallinity (70%) and remarkable barrier properties. The permeability of CNF to oxygen at 23°C and 0% relative humidity (RH) is equal to 0.0009 cm^3^·mm/(m^2^·d·atm) (Aulin et al., 2010), which is approximately 3–20 times lower than that of ethylene vinyl alcohol [0.0024-0.02 cm^3^·mm/(m^2^·d·atm) (Woishnis, 1995; Zhang et al., 2001)] and more than 1000 times lower than that of polyethylene terephthalate (PET) [0.91 cm^3^·mm/(m^2^·d·atm) (Woishnis, 1995; Aulin et al., 2010, 2012; Lavoine et al., 2012)] under the same conditions. Such a remarkable barrier performance is however compromised by the presence of moisture, which disrupts the H-bonded network and swells the amorphous zone between the fibrils, leading to a considerable increase of oxygen and water vapor permeation for RH values above 50–60% (Aulin et al., 2010). Several strategies to reduce the serious lack of hygroscopic stability of CNF have been proposed (Lu et al., 2008; Nystrom et al., 2009; Svagan et al., 2009; Rodionova et al., 2011; Spence et al., 2011; Ho et al., 2012; Lavoine et al., 2012; Aulin and Strom, 2013; Sharma et al., 2014). Thermal treatments at temperatures in the range from 100°C to 175°C for several hours were found to reduce the permeability of CNF to oxygen and water vapor due to increase in crystallinity and reduction of the inter fibril space or porosity (Sharma et al., 2014). The introduction of layered silicates in a trimethylammonium-modified nanocellulose matrix was proposed to create a tortuous path for the diffusing gas, and the resulting water vapor permeability was a factor of 30 times lower than that of plain paper (Ho et al., 2012). Various chemical modifications of the hydrophilic fibrils to render CNF hydrophobic were also demonstrated, including acetylation (Rodionova et al., 2011), silylation (Lu et al., 2008), and fluorination (Nystrom et al., 2009). A further route was in the form of composites and multilayers of CNF with various hydrophobic moieties. Examples include multilayer coatings of nanocellulose and alkyd resins on paper (Aulin and Strom, 2013), protective coatings such as beeswax, paraffin, starch and shellac, or composites with mineral fillers such as kaolin clay and calcium carbonate added to microfibrillated cellulose (Svagan et al., 2009; Hult et al., 2010; Spence et al., 2011). These approaches to suppress the sensitivity of CNF to moisture uptake required time consuming drying, and their efficiency was often limited, for instance due to poor interfacial interactions in the case of composites with mineral fillers. To overcome this problem, CNF was for instance surface-modified with ceric ammonium nitrate for chemical bonding with a moisture stable, UV-curable acrylate (Galland et al., 2014). The moisture sensitivity of the oxygen permeability of the resulting composite was drastically reduced compared with CNF alone. Nevertheless, the actual barrier performance of the composite, with an OTR of few cm^3^/(m^2^.day.bar) remained far from the targets previously mentioned for flexible electronics.

The objective of the present work was to develop multilayer films based on CNF substrates and organic-inorganic hybrid multilayer coatings with lower permeability to water vapor and to oxygen compared with that of uncoated CNF, especially in a humid environment, and generate a range of diffusion barrier materials for applications including food packaging and encapsulation of solar cells. Organic-inorganic hybrids are molecular composite networks obtained through the integrative dual-cure synthesis of organic thermally- or photo-curable precursors and inorganic sol-gel precursors (Amrerg-Schwab et al., 1998; Haas and Wolter, 1999; Haas et al., 1999; Chang et al., 2009; Vaško et al., 2009; Geiser et al., 2012; Jancovicova et al., 2013). Owing to high network densities, the presence of specific functional groups and polarity levels, these hybrid materials were shown to offer good barrier performance and excellent adhesion to a wide variety of substrates. For instance, the oxygen permeability at 23°C and 50% RH of biaxially oriented polypropylene films coated with hybrid barriers was up to 50 times lower than that of the uncoated polymer film, and was further reduced by more than 600 times with the addition of a single SiO_x_ layer (Amrerg-Schwab et al., 1998). It was moreover found that the polar interactions with the organic polymer substrates such as PET (Amrerg-Schwab et al., 1998) or the condensation reaction of the sol with hydroxyl groups at the surface of various substrates such as surface treated polyester (Chou and Cao, 2003), glass (Kron et al., 1994), silicone (Ochi et al., 2001), and AlO_x_ (Miesbauer et al., 2014) led to strong physical and covalent bonds at the interface, hence high adhesion levels.

The organic phase provides elasticity and toughness to the network while the inorganic phase provides thermal stability and contributes to prevent the permeation of small molecules due to increasing tortuosity of the diffusion path. It was moreover foreseen that these hybrids would act as smooth planarization layers to reduce the roughness and associated light scattering, and thus improve the transparency of CNF films. The hybrids would also provide universal, hard surfaces for further deposition of high quality layers. More interestingly, it was anticipated that their permeability would be low enough so that it would be possible to achieve ultrahigh barriers with reduced the number of inorganic layers in the multilayer stack.

Focus of the work was on epoxy hybrids (Serra et al., 2016), in which the epoxy forms a dense network with a high glass transition temperature (T_g_), a low shrinkage and good adhesion to polar surfaces (Varma et al., 1997). However, the main challenge was related to the presence of water molecules during the formation of the sol-gel inorganic network as a result of hydrolysis and condensation of alkoxy-silanes and organo-alkoxy-silanes. One the one hand the conversion of epoxy monomers is inhibited by water due to chain transfer reactions with the hydroxyl terminated chains. The resulting short, un-crosslinked chains act as plasticizers, which is detrimental to the properties of the cured hybrid (Belon et al., 2010). On the other hand, as mentioned previously, CNF is highly sensitive to moisture. Attention was therefore paid first to carefully control and optimize the dual-cure process conditions to overcome these moisture issues and create a dense hybrid network. Focus was then put to investigate the influence of the hybrid composition on the roughness, optical transparency, interfacial adhesion, water vapor and oxygen permeability of coated CNF films. The creation of multilayers including additional inorganic layers formed by plasma-enhanced chemical vapor deposition (PECVD), and the resulting improvement of barrier performance was finally investigated.

## Experimental

### CNF films

Two different generations of CNF films were used as substrates. The first generation (GEN1) was prepared by enzymatic pre-treatment (Pääkko et al., 2007) of fully bleached Kraft pulp from softwood followed by a three-pass homogenization in a microfluidizer (Microfluidics Corp., United States) at 1200 bars. 15 wt% sorbitol was added to the CNF dispersion to reduce brittleness and increase strain at break. The films were subsequently produced with a slit coater by coating a 2.5 wt% solids dispersion on a polished stainless steel sheet attached to an aluminum plate heated at 95°C. After casting the CNF film was left to dry for 5 min prior to being released. The thickness of the GEN1 films was 41 μm. It was observed that the air-side was rougher than the side in contact with the smooth metal surface (Table 1). The second generation (GEN2) was a commercial never-dried, totally chlorine-free bleached softwood sulphite dissolving pulp (Domsjö Fabriker AB, Sweden) comprising 40 wt% Scots Pine (*Pinus sylvestris*) and 60 wt% Norway Spruce (*Picea abies*) with a hemicellulose content of 4.5 wt% (measured as solubility in 18% NaOH, R18) and a lignin content of 0.6 wt%. The anionic CNF was prepared by using a carboxymethylation pre-treatment of the pulp fibers (Wågberg et al., 2008), as detailed in the Supplementary Information. This pre-treatment included an impregnation step of the fibers with a NaHCO3 solution (4 wt% solution) in order to convert the carboxyl groups to their sodium form to further enhance the delamination of the fibers into nanofibers. After this treatment, the fibers were passed through a high-pressure homogenizer (Microfluidizer M-110EH, Microfluidics Corp., United States). To ensure full delamination of the fibers into individual nanofibrils, six passes through the homogenizer were carried out, each with a subsequent dilution step. The final concentration of nanofiber dispersion was 0.2 wt%. Such a procedure leads to the liberation of cellulose I nanofibers, mostly with cross-sectional diameters of 5–20 nm and lengths of a few micrometers. The total charge density of the highly carboxymethylated CNF dispersion was measured to be 627 meq/g by conductometric titration. This corresponds to a degree of substitution of 0.1. Free-standing films were solvent casted at 23°C and 50% RH in square, 23.3 × 23.3 cm polycarbonate boxes. The targeted film grammage was 20 g/m^2^ and the thickness of the GEN2 films was 19 μm. Again, the air-side was rougher than the side in contact with the smooth plastic surface. In order to decrease the moisture content in the CNF before further coating operations, all CNF films were dried at 100°C for 24 h under vacuum. Such process was reported to crosslink the nanocellulose fibrils and improve CNF stability vs. possible diffusion of water and alcohols (Sharma et al., 2014).

**Table 1:** Coating thickness *h*_*c*_, RMS roughness R_RMS_, optical transmission at 550 nm T_550_, WVTR, OTR, and corresponding permeability values of bare CNF, coated CNF and coatings.

**Substrate**	**Coating^a^**	***h_*c*_* (μm)**	**R${}_{\text{RMS}}^{\text{b}}$ (nm)**	**T_550_ (%)**	**WVTR^d^ (g/m^2^/day)**	***P_*H*2*O*_* (g.mm/m^2^/day/atm)**	***P_*H*2*O, c*_* (g.mm/m^2^/day/atm)**	**OTR^e^ (cm^3^/m^2^/day/bar)**	***P_*O*2_* (cm^3^.mm/m^2^/day/bar)**	***P_*O*2, *c*_* (cm^3^.mm/m^2^/day/bar)**
GEN1 (41.4 μm)	–	–	8.70 ± 1.72/13.3 ± 3.44^b^	10.2	13.60 ± 0.42	17.2	–	< 0.008/10.83 ± 0.99	< 0.0003/0.45	–
	Pure epoxy	26.1	0.24 ± 0.01	34.6	5.76 ± 0.58	11.9	7.96	< 0.008/5.77 ± 0.87	< 0.0004/0.40	< 0.0002/0.36
	0T5G	32.1	1.37 ± 0.07	–	4.86	10.9	7.41	–	–	–
		2.2	–	–	8.89	11.8	1.71	–	–	–
	SiN_x_/0T5G	0.15/33.0	–	–	0.70 ± 0.1	1.62	< 0.76	–	–	–
	10T10G	21.5	0.57 ± 0.12	–	5.80 ± 0.42	11.1	6.64	–	–	–
	10T20G	36.8	1.17 ± 0.33	32.1	4.54 ± 0.24	10.8	7.69	–	–	–
		21.8	–	–	4.92	9.50	5.14	–	–	–
		2.2	1.20 ± 0.62	34.3/66.0^c^	8.98 ± 0.33	11.9	1.48	< 0.008/8.05 ± 0.07	< 0.0004/0.35	2 × 10^−6^/0.08
	SiN_x_/10T20G	0.15/3.0	–	–	< 0.02	< 0.028	< 0.002	–	–	–
	30T10G	25.3	0.57 ± 0.16	30.9	5.89 ± 0.24	12.0	8.03	–	–	–
	40T10G	33.5	2.03 ± 0.06	27.8	5.80	13.3	10.4	–	–	–
GEN2 (19.4 μm)	–	–	4.73 ± 1.07/6.77 ± 2.57^b^	69.6	65.20 ± 2.33	38.6	–	< 0.008/9.13 ± 0.19	< 0.0002/0.18	–
	Pure epoxy	33.4	0.29 ± 0.02	72.2	7.09	11.4	8.12	< 0.008/3.04 ± 0.03	< 0.0004/0.16	< 0.0003/0.15
	10T20G	27.0	–	–	9.50 ± 0.57	13.5	9.19	–	–	–
		4.7	0.967 ± 0.12	78.2/87.0^c^	16.10	11.9	3.10	< 0.008/5.66	< 0.0002/0.14	4 × 10^−5^/0.07

*The coating permeability values P_H_2_O, c_ and P_O2, c_ were calculated using Equation 2 from the known coating and substrate thicknesses and substrate permeability values*.

a *Nomenclature “xTyG” of hybrid coatings: x and y stand for the initial wt% of TEOS and hydrolyzed wt% of GPTS, respectively (the remaining percentage was epoxy resin with 3 wt% of photoinitiator with respect to total formulation)*.

b *RMS roughness on mold side (smooth plastic or metal)/air side*.

c *single side/double side coated CNF*.

d *at 38°C, 50% RH (g/m^2^/day). The values given without standard deviation were measured in single chamber*.

e *at 23°C, RH below 50%/at 23°C, 70% RH (cm^3^/m^2^/day/bar). The values given without standard deviation were measured in single chamber*.

Notice that the peak decomposition temperature of GEN1 and GEN2 CNF films was approximately 340°C and 300°C, respectively, irrespective of the atmosphere conditions (see Figure S1), so that the films were thermally stable for all coating processes, in particular the PECVD of the inorganic films.

### Hybrid coatings

**Scheme 1:**
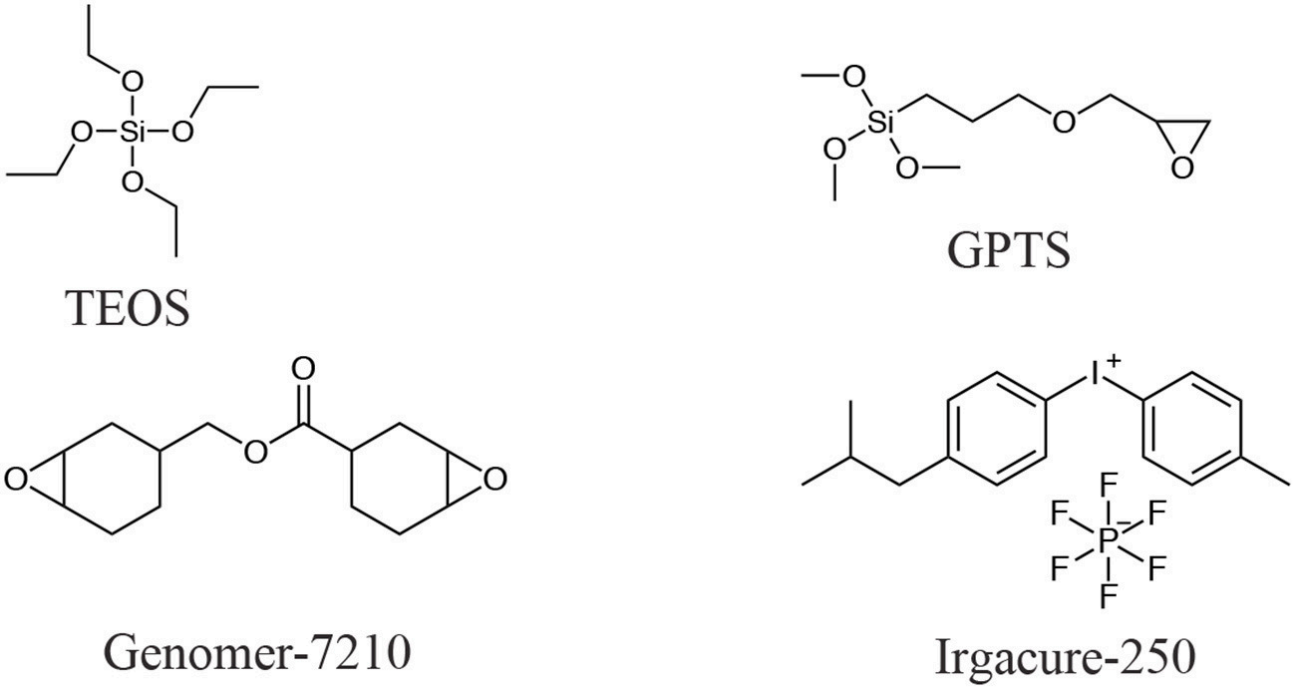
Chemical structures of the compounds used in this work.

Hybrid coatings were produced using a dual-cure, condensation-photopolymerization process. The chemical structures of the material precursors are shown in Scheme 1. A cycloaliphatic epoxy resin (Genomer-7210, Rahn, Germany) was used to form the UV-cured organic network. Iodonium, (4-methylphenyl) [4-(2-methylpropyl) phenyl]-, hexafluorophosphate (Irgacure-250, BASF, Switzerland) was used as cationic photoinitiator. Tetraethylorthosilicate (TEOS, Aldrich, United States) was used as the inorganic precursor to form the sol-gel siloxane network. In order to induce bonding between the organic and the inorganic phases, the coupling agent 3-glycidoxypropyltrimethylenesilane (GPTS, Aldrich, United States) was added to the formulations. HCl (1 N solution in water, Aldrich, United States) was used as a catalyst to provide acidic conditions for the sol-gel process. Ethanol (EtOH, 96%, ABCR, Germany) was used as a solvent for solubilization of water in the alkoxysilane.

A first step was dedicated to optimize the dual-cure process sequence, in particular to avoid the presence of problematic water molecules. When photopolymerization was performed prior to sol-gel reactions, phase separation between TEOS and crosslinking epoxy was observed after 10 s of UV irradiation, leading to hazy or even opaque coatings when thickness was above 50 μm. Transparent coatings were obtained when photopolymerization was started sometime after thermal condensation had been initiated. However, the degree of conversion of these hybrid coatings was reduced due to the presence of moisture during hydrolysis and the WVTR of these coatings was too high for practical use. Moreover, the duration of the dual-cure process was several hours, which was not suitable for technologies which require very short curing times such as roll-to-roll processes. The solution was to pre-hydrolyze and partly condense the inorganic precursor and coupling agent, prior to further polymerization with the epoxy. In fact, the sol-gel reaction kinetics is mainly controlled by temperature and relative humidity. A high level of humidity favors hydrolysis and is beneficial to the formation of the siloxane network. The pre-hydrolysis and partial condensation of TEOS and GPTS were performed separately to avoid phase separation between both due to different hydrolysis rates. Further details are provided in the Supplementary Information. The TEOS and GPTS solutions were subsequently mixed together and the epoxide monomer and photoinitiator were added, the latter at a concentration of 3 wt% with respect to the total formulation. The formulations were adapted to set the ratio of initial TEOS content to be between 0 and 40 wt% and hydrolyzed GPTS between 0 and 20 wt%. The liquid formulations were applied onto the smooth side of CNF substrates under atmospheric conditions using wire-wound applicators, to achieve controlled coating thickness in the range 2–40 μm. The coatings were cured with a 400 W metal halide lamp (Dymax 2000-EC, United States) at an intensity on the surface of the sample of 60 mW/cm^2^ for 2 min. The light intensity was measured using a calibrated radiometer (Silver Line, CON-TROL-CURE, Germany), between 230 and 410 nm. The coated CNF samples were annealed at 100°C for 24 h under vacuum to re-dry the CNF substrate and post-cure both organic and inorganic networks. The total thickness of each sample was measured on at least 10 positions using a digital micrometer (Mitutoyo, Japan) with a 1 μm resolution and average values were reported. A total of five hybrid formulations were prepared and will be referred to in the following as xTyG, where x and y stand for the initial wt% of TEOS and hydrolyzed wt% of GPTS, respectively. The remaining percentage was epoxy resin with 3 wt% of photoinitiator with respect to total formulation. Plain epoxy coatings based on the same Genomer-7210 and including 3 wt% of photoinitiator, cured with the same conditions as for the hybrid coatings were also produced as reference.

### Inorganic layers

Thin silicon nitride films (SiN_x_) were deposited by plasma enhanced chemical vapor deposition (PECVD) in a parallel plate radio frequency plasma reactor (Oxford Instruments) (Van de Weijer et al., 2017) on 10T20G hybrid coated GEN1 CNF films. The SiN_x_ film thickness was 150 nm.

### Characterization methods

Attenuated total reflectance Fourier transform infrared spectroscopy of the initial and pre-hydrolyzed inorganic precursor was carried out (ATR-FTIR Thermo Fisher Scientific Nicolet 6700, United States) equipped with a ZnSe crystal ATR unit. The spectra were acquired with 32 scans and a resolution of 4 cm^−1^ in the range of 4000–650 cm^−1^. The long-term stability of the hybrid formulations was determined from their viscosity, measured with a rheometer (AR 2000, TA Instruments, United States), using a parallel plate configuration with 25 mm diameter plates. The 250 μm thick specimens were subjected to a dynamic shear strain with an amplitude of 1% and the frequency was varied from 0.01 to 100 Hz. The tests were carried out at 23°C on fresh samples, and on samples stored either at 5°C or at 25°C for different times up to 6 weeks. Optical transmission measurements of bare and coated CNF films were performed using a UV-Vis spectrophotometer (Jasco V-670, Germany). The surface morphology of the same materials was characterized by atomic force microscopy (AFM, NanoScope IIIa, Veeco, United States) in tapping mode using an Ultrasharp NSC16/No Al cantilever. The lowest measurement range and resolution of the AFM were 1 μm and 0.3 nm, respectively. The root mean square (RMS) roughness, defined as the RMS average of the height deviations from the mean value was measured on 3 randomly selected, 1 × 1 μm^2^ areas. Thermogravimetric analyses of the CNF films were carried out under air and nitrogen (Q5000, TA Instrument, United States) during heating from 105°C to 600°C at a rate of 10°C/min. Samples were exposed first to an isothermal drying step at 105°C for 20 min to remove moisture before heating. The elastic modulus and glass transition temperature of bare and coated GEN1 CNF with epoxy and hybrid coatings were determined by dynamic mechanical analysis (DMA Q800, TA Instruments, United States). Rectangular samples of bare CNF, 30 μm thick epoxy/hybrid coated GEN1 CNF and 250 μm thick, standalone epoxy/hybrid films, all with a width of 5 mm and a gauge length of 11 mm were characterized. The standalone films were produced by pouring the liquid formulations into silicone molds and UV-curing for 5 min from the top-side, demolding, turning samples upside down and further curing the other side for another 5 min. All DMA samples were tested in tensile configuration. They were first cooled down to 0°C, let equilibrate for 3 min and heated to 230°C at a rate of 5°C/min. The water vapor transmission rate, WVTR, of bare and coated CNF films was measured at 38°C and 50% RH using an electrolytic P_2_O_5_ sensor (Systech 7001, United Kingdom), with a measurement limit of 0.02 g/m^2^/day. For each experiment, two circular specimens were cut from the films and mounted in the two parallel chambers of the apparatus using a steel mask with a circular opening of 5 cm^2^. The two chambers were purged with nitrogen until baseline stabilization and the permeation test was initiated by exposing one side of the film to a flow of pure water vapor. The steady-state WVTR data were collected for both chambers and the mean values were calculated. Oxygen transmission rate (OTR) of bare and coated CNF films was measured at 23°C and either 50% RH or 70% RH by using a coulometric cell (Systech 8001, United Kingdom), with a measurement limit of 0.008 cm^3^/m^2^/day/bar, using a steel mask with a circular opening of 5 cm^2^. The chambers were purged with nitrogen until baseline stabilization and the permeation test was initiated by exposing one side of the film to a flow of pure oxygen gas (1 bar). The steady-state OTR data were collected for both chambers and the mean values were calculated. The permeability of bare and coated CNF films to water vapor (*P*_H2O_) and oxygen (*P*_O2_) was calculated from the respective transmission rate as:

(1) $$\begin{array}{r} P_{H2O}=WVTR\cdot h/\Delta p\;and\;P_{O2}=OTR\cdot h \end{array}$$

where *h* is the thickness of the film and Δ*p* is the water vapor pressure at the selected temperature and relative humidity (Aulin and Strom, 2013). The permeability of the coating itself, *P*_*c*_, to either water vapor or oxygen was derived from the known permeability of the CNF substrate, *P*_*s*_, and thickness of both substrate, *h*_*s*_, and coating, *h*_*c*_, assuming a parallel-type permeation behavior:

(2) $$\begin{array}{r} P_{c}=\frac{h_{c}}{\frac{h}{P}\;-\;\frac{h_{s}}{P_{s}}} \end{array}$$

The morphology of the samples surface was characterized using scanning electron microscopy (SEM, Gemini-SEM 300, Zeiss, United States). A 6 nm thick iridium layer was sputtered onto the samples prior to the SEM analyses. The interface between the CNF and the coatings was also characterized from cross-sections using SEM. Samples were embedded in a resin (Epon 812, Hexion inc., Ohio, United States), cured for 24 h at 60°C and stored under vacuum to prevent water absorption of CNF. The cross-sections of embedded samples were prepared by ultramicrotomy (EM UC7, Leica, Germany) at ambient temperature. A first cut was performed with a razor blade to obtain the raw surface. The main trim was carried out with a Cryotrim 45° diamond knife (Diatome, United Kingdom) at speed of 20 mm/s and a feed step of 500 nm, and an ultra 35° diamond knife (Diatome, United Kingdom) with a speed of 0.6 mm/s and a feed step of 200 nm. During all these operations, the state of the surface was observed with an optical microscope, which revealed the apparition of delaminations within the layered structure of the CNF substrate, as will be shown in the following section.

## Results and discussion

The composition and properties of the bare and coated CNF films (thickness, roughness, optical transmission and permeability to water vapor and to oxygen) are reported in Table 1. Additional data (FTIR, rheology and DMA) are detailed in the Supplementary Information File.

### CNF substrates

**Figure 1:**
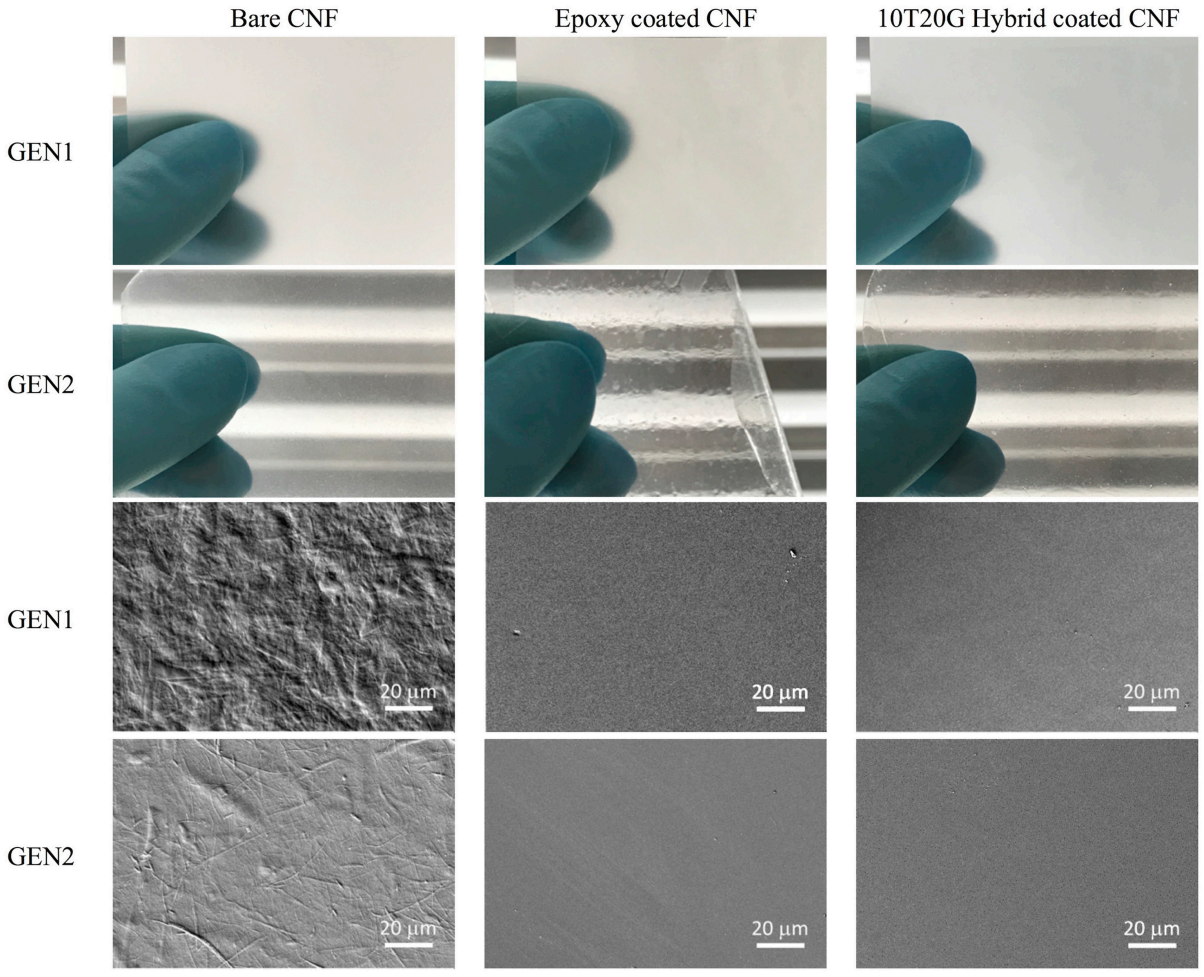
Photographs of bare and coated GEN1 and GEN2 CNF substrates (top two rows, sample size approximately 2 × 4 cm, coating thickness ~10 μm, the photographs were taken using a corrugated metal background for improved image contrast) and corresponding surface topography obtained by SEM (bottom two rows; smooth metal side for bare GEN1 and smooth plastic side for bare GEN2 substrates). The 10T20G hybrid is based on initial concentrations of 10 wt% of TEOS, 20 wt% of hydrolyzed GPTS and 70 wt% of epoxy including 3 wt% of photoinitiator).

Figure 1 shows the visual appearance and surface morphology of the two generations of bare and coated CNF films. It is evident that GEN1 films were very hazy with low optical transmission, a result of their relatively large surface roughness. In contrast GEN2 films were clear and smoother, their roughness being two times lower and optical transmission at 550 nm almost 7 times higher compared with GEN1 films. The carboxymetylation treatment for the GEN2 CNF combined with multiple high-pressure homogenization steps enhanced delamination of cellulose fibers into individual monodispersed nanofibers, resulting in a narrow fibril size distribution and hence a lower surface roughness and greatly improved transparency (Aulin et al., 2010). The properties of the coated films also shown in Figure 1 are detailed in the following sections. The storage modulus of the GEN1 CNF was found to be equal to 10.8 GPa at 25°C as detailed in the Supplementary Information, which is remarkably much higher than that of most synthetic polymers. A broad transition in the mechanical behavior was found to occur around 190°C, which might be attributed to the amorphous part of cellulose fibrils.

The WVTR at 38°C and 50% RH of GEN1 CNF was 2 to 15 times lower than values reported for CNF produced by TEMPO-mediated oxidation or by mechanical treatment, and which were measured at 23°C and 50% RH (Rodionova et al., 2011; Kumar et al., 2014), and comparable to that of and low-density polyethylene and polyethylene terephthalate films. These two synthetic polymers have similar WVTR in the range 10–14 g/m^2^/day at 38°C and 90% RH when normalized to the same 41 μm thickness as that of the CNF film. The WVTR of the GEN2 film was five times higher, due to its reduced thickness but also to its water vapor permeability calculated using Eq. 1 and found to be twice as high compared to the GEN1 film. The high surface charge of the carboxymethylated CNF resulted in considerable moisture uptake and hence moisture-induced swelling of the films, which in turn increased its permeability to water vapor and oxygen, to a much larger extent compared with neutral GEN1 CNF (Belbekhouche et al., 2011). In addition, it was observed that the rod-like fibril morphology of carboxymethylated CNF led to a higher permeability compared to microfibrillated cellulose made of more flexible fibers (Kumar et al., 2014).

The OTR of both bare and coated GEN1 and GEN2 CNF substrates at 23°C was below the detection limit of the instrument (0.008 cm^3^/m^2^/day/bar) when the RH was below 50%, as previously reported (Aulin et al., 2010). In these ambient conditions CNF is indeed far less permeable to oxygen than LDPE (permeance of 100 μm thick films equal to 2200 cm^3^/m^2^/day/bar; Leterrier, 2003) and PET (OTR of 36 μm thick films equal to 36.6 cm^3^/m^2^/day/bar; Vaško et al., 2009). The permeance of the CNF films became measurable with increasing RH, and found to be close to 10 cm^3^/m^2^/day/bar at 70% RH for both 41 μm thick GEN1 and 19 μm thick GEN2. The oxygen permeability of GEN2 CNF was thus 2.5 times lower than GEN1 CNF. This result is in contrast with the finding of a higher water vapor permeability for the GEN2 CNF and reflects the different transport behavior between oxygen and polar water molecules (Crank and Park, 1968; Belbekhouche et al., 2011). As for water transport, the combination of high surface charge and fibrillar nature of GEN2 CNF promoted swelling of the material upon exposure to moisture, which enhanced both the sorption of water molecules, and their transport within the network of short nanofibrils, in comparison with less polar GEN1 CNF. The unexpected reduced oxygen permeability in GEN2 CNF compared with GEN1 CNF was attributed to an overall higher cohesive energy density and more specifically closer packed structure of the carboxymethylated nanocellulose owing to the improved delamination of pulp into small nanofibrils. In addition, oxygen molecules have a much larger kinetic diameter (3.46 vs. 2.65 Å for water), which restricted their diffusion within the densely packed nanofibril network.

### Epoxy coated CNF films

**Figure 2:**
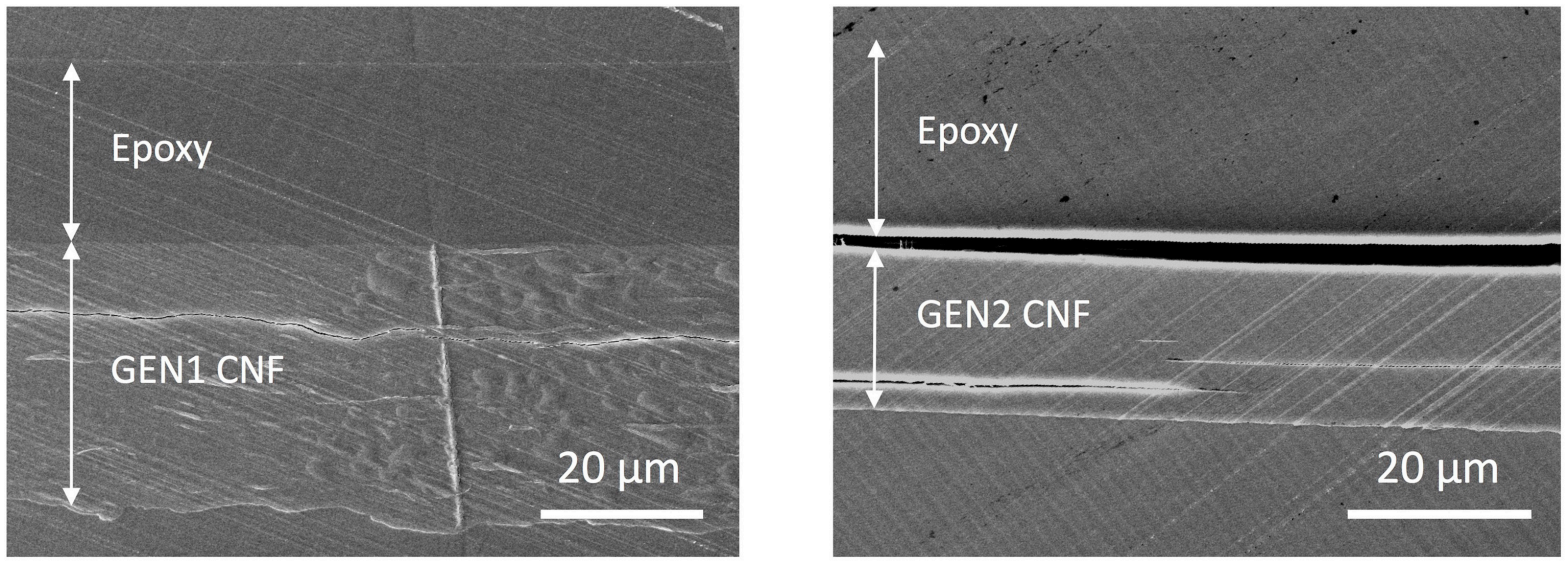
Electron micrographs of the cross-sections of epoxy coated GEN1 **(Left)** and GEN2 CNF **(Right)** substrates.

Pure epoxy coatings were produced as reference. It turned out that the epoxy monomer did not wet well both CNF substrate generations, and this effect was more pronounced for the GEN2 case. In that case small holes and visually apparent macro-roughness on the cured coatings when the thickness was below 20 μm were evident (Figure 1). These defective coatings did not enable any improvement in barrier performance of the CNF substrates. Figure 2 shows the cross section of the epoxy coated CNF of both generations. In-plane cracks were visible within the two types of CNF substrates, which were artifacts of the sample preparation process as detailed in the Experimental Section, and in fact revealed the process-induced layered nature of the films (Aulin et al., 2010). The interface between the epoxy and the GEN1 CNF was in contrast intact. The fact that it was not damaged during sample preparation provides an indication of rather good adhesion. However, the epoxy coating was totally delaminated from the GEN2 substrate, for which the wettability issue was more pronounced. Nevertheless, the application of thicker layers, around 30 μm led to defect-free coatings on large enough areas suitable for permeation tests.

The DMA data of the epoxy (30 μm) coated GEN1 film is detailed in Figure S4. Its storage modulus was found to be equal to 6.5 GPa, again, a rather high value for organic materials. This 40% drop compared with the bare film was attributed to the lower modulus of the epoxy coating, found to be equal to 2.6 GPa using standalone, 250 μm thick films. Using this value for the epoxy coating and applying the rule of mixture led to a coated CNF modulus of 7.3 GPa, i.e., 12% higher than the measured value of 6.5 GPa. The T_g_ of the epoxy coating was moreover found to be equal to 155°C.

The RMS roughness of the epoxy coatings on GEN1 CNF was found to be 0.24 nm, considerably lower than that of the bare GEN1 CNF. This planarization effect was remarkable, with a roughness among the lowest values reported in previous works (Fahlteich et al., 2013), and usually found to be around 0.6–0.7 nm (Leterrier et al., 2004; Yan et al., 2005). In spite of visual heterogeneities, the epoxy coating improved the optical transmission of this CNF film more than 3-fold. It also decreased more than 2-fold the WVTR of the GEN1 CNF, to values close to 5 g/m^2^/day, that can be considered as high barrier for food packaging (Hult et al., 2010). The permeability to water vapor of the epoxy coating itself calculated using Equation 2 was close to 8 g.mm/m^2^/day/atm, i.e., approximately two times lower than that of the CNF substrate. The roughness of the epoxy coating on GEN2 CNF was found to be almost identical to that reported above in case of GEN1 CNF, which means that epoxy acted as a planarization coating. The good transparency of GEN2 CNF was barely improved by the epoxy. The WVTR of the GEN2 CNF was reduced by almost an order of magnitude after coating with the epoxy. This considerable improvement was in fact the result of the higher thickness and lower permeability of the epoxy with respect to the GEN2 CNF. Notice that the water vapor permeability of the epoxy on both types of CNF was the same as reported in Table 1. This result implies that the structure of the epoxy was not influenced by the nature of the CNF surface, despite a different wetting behavior.

The OTR values of epoxy coated GEN1 and GEN2 CNF substrates were 2 and 3 times lower than that of the bare substrates, respectively. Surprisingly, in contrast with the case of water, the oxygen permeability of the epoxy coating was different between the two types of CNFs. This finding is puzzling and was argued to result from differences in wetting and interfacial structures, combined with the known differences in diffusion and solubility behavior between oxygen and water in polymers (Belbekhouche et al., 2011).

To summarize, the application of an epoxy coating considerably improved the water vapor and oxygen barrier performance of the two types of CNF, to values suitable for food packaging, with large improvement in transparency and an excellent planarization effect. These improvements were, however, offset by a poor wetting and lack of adhesion of the epoxy, especially in case of the GEN2 substrates. This drawback came in addition to local heterogeneities of the epoxy, which would severely compromise the use of such coated substrates for application of ultrahigh barrier multilayers.

### Hybrid coated CNF films

FTIR was used to monitor the progress of the pre-hydrolysis step as detailed in Figure S2 and confirmed the decrease of ethoxy functions of TEOS and methoxy functions of GPTS, the formation of Si-OH groups after water and ethanol evaporation, and the formation of Si-O-Si bonds due to condensation reactions. It was also found that hydrolysis proceeded without causing the opening of the epoxy ring (Shajesh et al., 2009; Peng et al., 2012). The long-term stability of the formulations was assessed from the evolution of their complex viscosity as detailed in the Supplementary Information File. It was found to mostly depend on the concentration of TEOS (Figure S3). The formulation with lower amount of TEOS (10T20G i.e., 10 wt% of TEOS and 20 wt% of hydrolyzed GPTS, and 70 wt% of epoxy including 3 wt% of photoinitiator) was stable up to 4 weeks of storage at both 5°C and 25°C. In contrast, the formulation 30T10G gelled after 1 week at 5°C, and after a day at 25°C, as clear result of the thermally activated condensation reaction, which was more pronounced for higher TEOS concentration.

**Figure 3:**
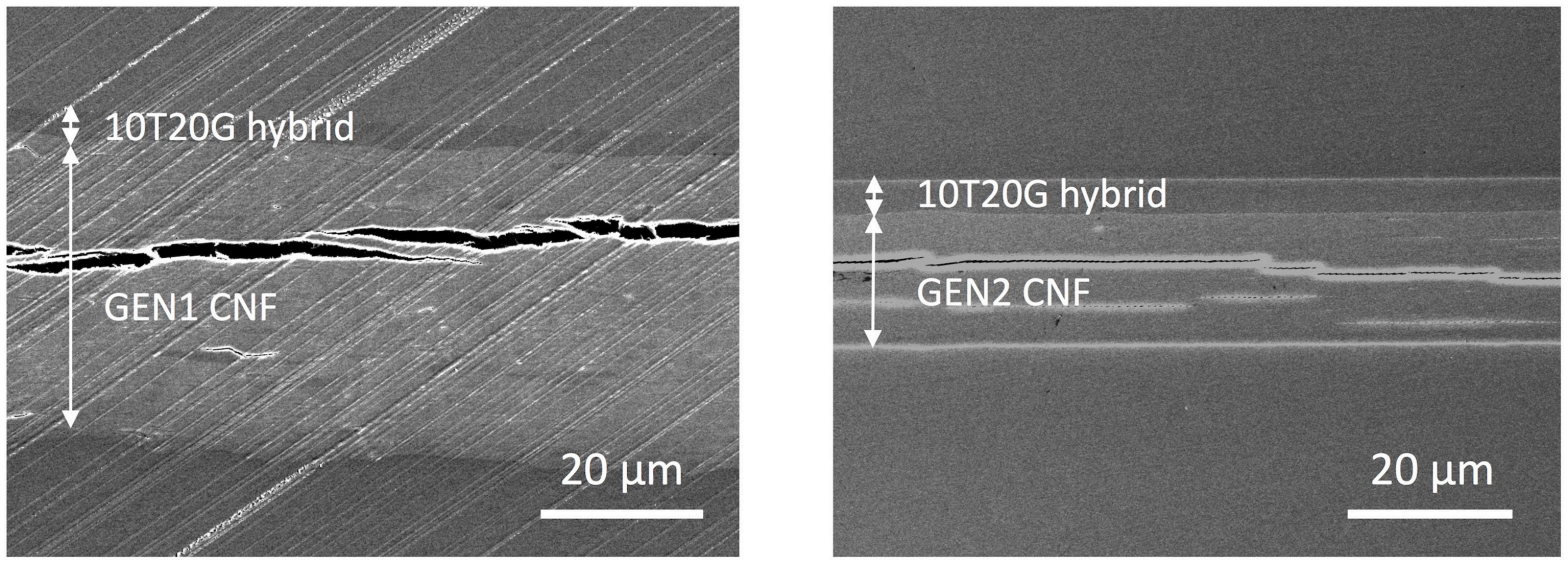
Electron micrographs of the cross-section of 3 μm thick 10T20G coated GEN1 **(Left)** and GEN2 CNF **(Right)** substrates. The 10T20G hybrid is based on initial concentrations of 10 wt% of TEOS, 20 wt% of hydrolyzed GPTS and 70 wt% of epoxy including 3 wt% of photoinitiator).

TEOS-based formulations were expected to wet CNF due to the affinity between the Si-OH groups of the hydrolyzed precursor and the OH-terminated CNF surfaces, and to strongly adhere to these surfaces through the formation of C-O-Si bonds (Haas et al., 1999). Figure 3 shows the cross section of 3 μm thick 10T20G hybrid coated GEN1 and GEN2 CNF. Again, in-plane delamination within the layered CNF structure was evident, which was an artifact of the sample preparation as previously pointed out. In contrast, the interface between the hybrid coatings and the CNF did not show any defect, which implies that the adhesion was high enough to prevent delamination to occur during sample preparation.

Wettability was further improved by adding 5% of GPTS to the formulation, leading to a reduced number of visually detectable defects and holes in the hybrid coating compared with the pure epoxy. Similarly, by increasing inorganic precursor ratio in the composition, very good wettability was obtained on both substrates and an adherent interface was observed for both cases.

The storage modulus of the 10T20G hybrid (30 μm) coated GEN1 CNF at 25°C was found to be equal to 5.6 GPa (see detailed data in Supplementary Information File, Figure S4). The Young's modulus of a 250 μm thick 10T20G hybrid sample at the same temperature was found to be equal to 2.05 GPa. This value is lower than that of the pure epoxy, most likely due to lack of condensation and formation of linear epoxy-siloxane chains, therefore reduced crosslink density in such thick hybrid coatings (Piscitelli et al., 2013), which will be discussed in details in the following. The application of the rule of mixture leads to a modulus of 7.1 GPa, which is 27% higher than the measured value. Such discrepancy could not be explained by the limited accuracy of the rule of mixtures (the application of more accurate classical laminate theory would in fact increase the discrepancy). It could if the modulus of the CNF substrate had decreased upon coating with the hybrid formulation, for instance due to moisture-induced plasticization effects. Additional work would be required to clarify this point. The T_g_ of the 10T20G hybrid was found to be equal to 150°C, i.e., 5°C lower than that of the pure epoxy.

The RMS roughness of the hybrid coatings on GEN1 CNF was found to be in the range 0.6–2 nm, independent of coating composition, i.e., up to 15 times lower compared with the bare substrate. It was equal to 0.97 nm on GEN2 CNF, a 5 times decrease. Such planarization effect warrants the application of high quality inorganic barrier films, as previously reported (Affinito et al., 1996; Coclite and Gleason, 2012; Fahlteich et al., 2013) and confirmed in the following section. Optical transparency was also considerably improved with the hybrid coatings. The transmission at 550 nm of bare GEN1 CNF increased from 10.2 to 34% for one side coated, to 66% for double side coated films. For GEN2 CNF the transmission increased from 69.6% for the bare film to 78.2% for one side coated, to 87% for double side coated films. Increasing the concentration of inorganic phase in the hybrid formulation slightly reduced the transparency of single side coated GEN1 CNF films (32.1% for 10T10G, 30.9% for 20T10G and 27.8% for 40T10G). This was tentatively attributed to increasingly larger inorganic domains in the hybrid composite (Kim, 2011). This small degradation in optical performance could be compensated by decreasing the thickness of the hybrid coating as shown for the 10T20G case.

The WVTR values of the GEN1 CNF films with ~30 μm thick hybrid coatings were similar to that of pure epoxy. A closer look at the water vapor permeability of the hybrid coating itself, obtained using Equation 2, revealed a decrease with increasing TEOS fraction up to 30 wt%, and an increase at higher concentration, possibly due to the lower degree of sol-gel condensation under the same curing conditions. The WVTR of hybrid coatings of similar thickness on GEN2 CNF films was 7 times lower than that of the bare CNF substrate, but was a factor two higher than hybrid coated GEN1 CNF (see Table 1). The water vapor permeability of the investigated 10T20G hybrid on GEN2 CNF was indeed approximately 50% higher than that obtained on GEN1 CNF. This result is significant and is the consequence of the relatively low degree of silica crosslinking for thick hybrid in combination with different interphase interaction behavior due to lower OH group density on GEN2 substrate. Such influences were in fact rather marginal, compared with the very large, 5-fold decrease of water vapor permeability when the coating thickness was reduced from 30–40 μm to 3 μm. This was attributed to the thickness-dependent absorption of the UV radiation above 200 nm by hydrolyzed alkoxides leading to further silanol condensation (Leest, 1995; Innocenzi and Brusatin, 2003; Han et al., 2007) and to the faster diffusion-controlled evaporation of water and ethanol byproducts. These combined effects eventually led to an increased network density with decreasing thickness. This UV-induced condensation reaction was confirmed with the following experiment. A 5 μm thick, pre-hydrolyzed TEOS solution was applied on the GEN1 CNF film and either immediately exposed to UV light or kept in the dark. The sample exposed to UV irradiation solidified after 30 s whereas the one kept in the dark was only able to solidify after 5 min. The OTR of 2–5 μm thick 10T20G hybrid coated GEN1 and GEN2 CNF films was again not measurable at 50% RH or lower humidity levels. At 70% RH it was ~1.5 times lower than that of the bare CNF. The corresponding oxygen permeability of the hybrids were a factor of 5 and 2.5 times lower than that of the GEN1 and GEN2 bare films, respectively.

The result of greatly reduced permeability at low thickness is interesting to design ultrahigh multilayer barrier structures based on stacks of inorganic layers and organic interlayers. As mentioned in the Introduction organic interlayers reduce the severity of defects in inorganic layers and considerably increase the diffusion path within the stack. The key rule for achieving ultrahigh barrier performance is that the thickness of these defect-decoupling interlayers should be smaller than the typical offset distance between the defects in adjacent inorganic layers (Tropsha and Harvey, 1997; Kim et al., 2004; Greener et al., 2007). Thinner interlayers, with a low intrinsic permeability as obtained with the epoxy hybrids would enable to reduce the number of inorganic layers, hence the production cost in view of encapsulation of moisture sensitive devices.

### Hybrid multilayer coated CNF films

**Figure 4:**
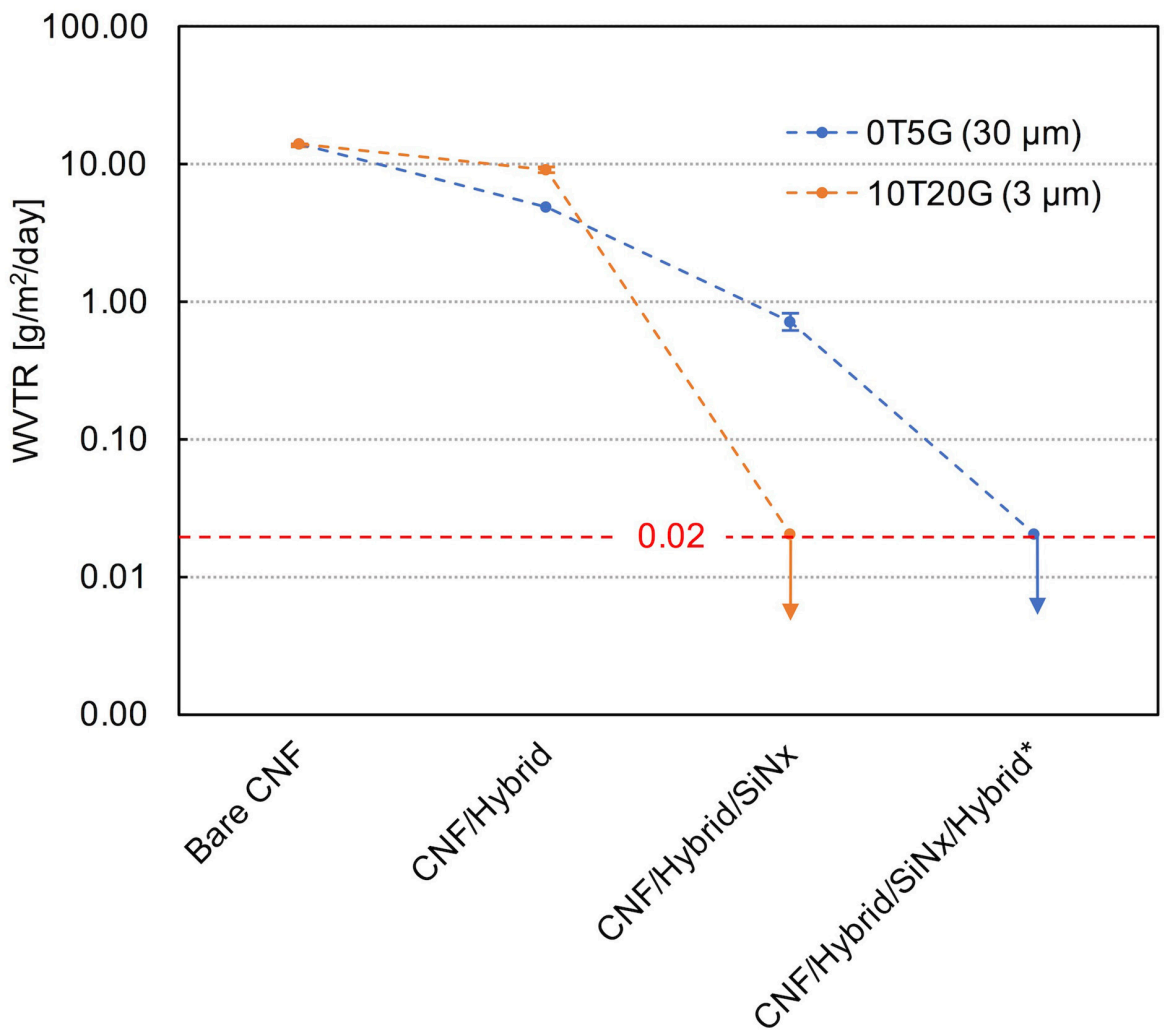
WVTR at 38°C and 50% RH of bare and coated GEN1 CNF, as indicated. The red dotted line corresponds to the detection limit of the permeation cell (0.02 g/m^2^/day). The nomenclature ‘xTyG’ for the hybrid coatings refer to initial concentrations of x wt% of TEOS and y wt% of hydrolyzed GPTS, the rest being epoxy including 3 wt% of photoinitiator. (^*^) The second hybrid layer coated on SiN_x_/0T5G is a 3 μm thick 10T20G.

Figure 4 shows the WVTR data of multilayer films based on hybrid and SiN_x_ layers on GEN1 CNF at 38°C and 50% RH, compared with the bare and hybrid coated CNF substrate. The WVTR of SiN_x_ (150 nm)/0T5G hybrid (40 μm) coated GEN1 CNF was found to be equal to 0.29 g/m^2^/day (23°C and 50% RH) and 0.7 g/m^2^/day (38°C and 50% RH). The inorganic layer thus enabled an order of magnitude decrease of the permeance of the hybrid coated CNF. These values are comparable with the WVTR of single SiO_x_ layer coated PET and polypropylene (PP), which range between 0.2–5 and 0.1–1 g/m^2^/day, respectively, at 23°C and 50% RH (Lange and Wyser, 2003), and of SiO_x_/Ormocer coatings on 36 μm thick PET (~0.7 g/m^2^/day at 23°C and 85% RH; Vaško et al., 2009) and on 20 μm thick PP (0.1 g/m^2^/day at 23°C and 50% RH; Lange and Wyser, 2003). Interestingly, the application of a second 10T20G hybrid layer (3 μm) on the SiN_x_/0T5G hybrid coated GEN1 CNF reduced the WVTR to below the detection limit of the electrolytic sensor (0.02 g/m^2^/day), both at 23°C and 38°C, and 50% RH. In this case, the improvement in WVTR was more than 35 times, that is much more than previously reported barrier improvements of ~10–30 times using similar hybrid configurations (Vaško et al., 2009). The WVTR reduction achieved with this second 10T20G hybrid layer was also much larger than that obtained with the first 10T20G hybrid. This important result imply that the liquid hybrid formulation healed defects in the inorganic layer (Vaško et al., 2009).

More, and quite remarkably, a single layer of SiN_x_ (150 nm) applied on the 10T20G hybrid (3 μm) coated GEN1 CNF immediately resulted in a decrease of WVTR below the detection limit (0.02 g/m^2^/day), an improvement of more than 680 times. This is significantly better than previously reported improvement factors obtained with other combinations of single inorganic films and planarization layers on synthetic polymer substrates, and found to be in the range of 100–400 (Affinito et al., 1996; Vaško et al., 2009; Logothetidis et al., 2010; Coclite and Gleason, 2012). The present very low permeation rate obtained with a single PECVD inorganic film is reminiscent of values as low as 0.05 g/m^2^/day reported for SiN_x_ coated polyethylene naphthalate and polycarbonate in rather extreme environmental conditions, 85°C, 85% RH, and 38°C, 100% RH, respectively (Lin et al., 1998; Kim et al., 2007). Notice the very large, more than 35 times difference in WVTR between the SiN_x_ coated 0T5G (30 μm) and 10T20G (3 μm). This difference results from the much lower water vapor permeability, and the improved interfacial bonding of the latter hybrid with the nitride layer due to its higher content of inorganic phase (Haas et al., 1999; Fahlteich et al., 2014).

The barrier performance toward ingress of water vapor of the present hybrid coatings with a single inorganic layer on CNF substrates is better than similar barrier coatings on synthetic polymer substrates. This performance, combined with good optical transparency and remarkable mechanical properties of biobased CNF should be useful for new packaging applications where environmental concern is the focus.

## Conclusions

UV curable organic-inorganic hybrid coatings based on epoxy and TEOS precursors were applied on enzymatically pre-treated and carboxymethylated CNF substrates, and further coated with PECVD SiN_x_ layers. The WVTR and transparency of CNF coated with pure epoxy were two times lower, and three times higher than that of the CNF, respectively, however epoxy did not properly wet the CNF surfaces and the interfacial adhesion was low. In contrast hybrid epoxy-silica coatings led to high adhesion levels owing to the formation of covalent interactions through condensation reactions, with similar barrier and optical performance compared with the pure epoxy. The hybrid coatings provided an excellent planarization effect, with roughness close to 1 nm, one to two orders of magnitude lower than that of the CNF substrates. Both WVTR and OTR values of the hybrid coated CNF were in the range 5-10 (g/m^2^/day for WVTR and cm^3^/m^2^/day/bar for OTR), which matches a large range of food and pharmaceutical packaging requirements. The permeability to water vapor of the hybrid coatings was found to decrease with increasing TEOS fraction up to 30 wt%, and increase at higher concentration, and also turned out to depend on their thickness, 3 μm thick coatings being five times less permeable than 30 μm thick coatings. This was due to due to further silanol condensation reaction by UV curing and faster evaporation of byproducts. The addition of a single 150 nm thick SiN_x_ layer on one of the hybrid coated CNF improved its barrier performance by more than 680 times, with WVTR below the 0.02 g/m^2^/day detection limit. It was moreover demonstrated that the barrier performance of these CNF based films was further improved by coating the inorganic layer with a second hybrid coating.

## Author contributions

FK and LM developed the hybrid coatings, did permeation and microstructural analyses. HR and MI developed the inorganic coatings and performed optical characterizations, GF, CA, and LA produced and characterized the CNF films, YL supervised the research work and reviewed the results. FK and YL wrote the paper.

### Conflict of interest statement

The authors declare that the research was conducted in the absence of any commercial or financial relationships that could be construed as a potential conflict of interest.
