# Supplementary material for: Organic-Inorganic Hybrid Planarization and Water Vapor Barrier Coatings on Cellulose Nanofibrils Substrates
Source: Front Chem. 2018 Nov 21;6:571. doi: 10.3389/fchem.2018.00571 (PMC6262297; doi:10.3389/fchem.2018.00571)
Supplement: Supplementary file 1 [file Data_Sheet_1.docx]

**SUPPLEMENTARY INFORMATION**

**Organic-Inorganic Hybrid Planarization and Water Vapor Barrier Coatings on Cellulose Nanofibrils Substrates**

F. Karasu^a^, L. Müller^a^, H. Ridaoui^b^, M. Ibn ElHaj^b^, G. Flodberg^c^, C. Aulin^c^, L. Axrup^d^, Y. Leterrier^a^*

^a^Laboratory for Processing of Advanced Composites (LPAC), Ecole Polytechnique Fédérale de Lausanne (EPFL), CH-1015 Lausanne, Switzerland

^b^Rolic Technologies Ltd., Gewerbestrasse 18, CH-4123 Allschwil, Switzerland

^c^RISE Bioeconomy, Drottning Kristinas väg 62, SE-114 86 Stockholm, Sweden

^d^Stora Enso Karlstad Research Centre, Sommargatan 101A, SE-656 37 Karlstad, Sweden

(*) corresponding author; yves.leterrier@epfl.ch

Figure 1S shows the thermogravimetric analyses of GEN1 and GEN2 CNF foils under N_2_ and air. Residual mass curves start with an approximately 3.5% and 5% weight loss for GEN1 and GEN2 foils, respectively, due to an isothermal drying run 105°C for 20 min performed before the heating run in order to remove the moisture content from the samples.

*Figure S1. Weight loss vs temperature and weight loss derivative of GEN1 and GEN2 CNF foils under N_2_ (left) and air (right).*

Figure 2S shows the FTIR spectra of TEOS and GPTS before and after hydrolysis. The decrease of ethoxy functions of TEOS after water and ethanol evaporation at 70°C under 310 mbar for 45 min is evident, with characteristic peaks at 793, 960, 1168 and 2974 cm^-1^. The broader peak at 960 cm^-1^ corresponds to the formation of Si-OH bonds. The formation of Si-O-Si bonds (condensation) is also evident with the emergence of broad bands at 1078 and 1163 cm^-1^. Methoxy functions of GPTS (821 and 2840 cm^-1^) disappeared after hydrolysis with a formation of Si-O-Si broad band around 1100 cm^-1^. The bands at 907 and 1255 cm^-1^ still exist after hydrolysis which indicates that hydrolysis proceeded without causing the epoxy ring opening.

|  |  |
| --- | --- |

*Figure S2. FTIR spectra of TEOS (left) and GPTS (right) before (solid lines) and after (dashed lines) hydrolysis.*

Figure S3 shows the complex viscosity at room temperature of hybrid formulations as a function of time of storage at 5°C and 25°C.

*Figure S3. Complex viscosity vs. time of hybrid formulations.*

Figures S4 and S5 show the tangent of the phase angle (ratio of loss modulus to storage modulus), and storage modulus from the dynamic mechanical analyses of bare GEN1 CNF, 30 µm thick epoxy and 10T20G coated GEN1 CNF, and 250 µm thick, standalone epoxy and 10T20G films, respectively.

*Figure S4. Tan δ (left) and storage modulus (right) of bare GEN1 CNF, 30 μm thick epoxy coated GEN1 CNF and 30 µm thick 10T20G coated GEN1 CNF substrates.*

*Figure S5. Tan δ (left) and storage modulus (right) of 250 µm thick epoxy and 10T20G stand-alone films after 10 min of UV curing (60 mW/cm^2^).*
